# Missed diagnosis of ankle pseudoaneurysm following ankle arthroscopy: a case report

**DOI:** 10.1186/1757-1626-2-162

**Published:** 2009-10-21

**Authors:** Ashok L Ramavath, Julie A Cornish, Muthu Ganapathi, Dean T Williams

**Affiliations:** 1 Department of Orthopaedic Surgery, Ysbyty Gwynedd Hospital, Penrhosgarnedd, Bangor, Gwynedd, UK, LL57 2PW, UK; 2 Department of Vascular Surgery, Ysbyty Gwynedd Hospital, Penrhosgarnedd, Bangor, Gwynedd, UK, LL57 2PW, UK

## Abstract

**Background:**

Pseudoaneurysm formation is rare complication after arthroscopy with incidence of 0.008%, easy to misdiagnose. Its potential catastrophic sequelae should not be underestimated.

**Case presentation:**

We present a case of missed diagnosis of traumatic anterior tibial artery pseudoaneurysm in a 39 years old female, instead treated as post operative arthroscopy infection. The diagnosis was confirmed with a duplex ultrasound scan and referred to the vascular surgeon with successful out come.

**Conclusion:**

In view of rare presentation this complication, it is easily missed. According to one study, incidence of anatomic variations of anterior tibial artery range from 2.4 to 12%. Because of this anatomical variation in course along with other factors, pseudoaneurysm formation at ankle is relatively high. In this report, we discuss the diagnosis, anatomical variations of anterior tibial artery and prevention of this complication following arthroscopy. We believe that surgeons operating in this region should take into account these anatomical variations preoperatively.

## Background

The use of ankle arthroscopy has increased over the last two decades. The procedure has a low morbidity, with vascular complications occurring in less than 1% of cases, especially when the anteromedial and anterolateral ports are used [1]. A sound anatomical knowledge of portals reduces the associated risk [2]. Anterior tibial artery pseudoaneurysm formation is a rare complication of ankle arthroscopy, with only six cases being described in the literature [1-5]. Due to the potential catastrophic sequelae of a pseudoaneurysm it is important for clinicians to have a high index of suspicion to prevent a missed or delayed diagnosis.

## Case presentation

A 39-year-old British Caucasian female in good general health with known history of rheumatoid arthritis presented to us complaining of recurrent bleeding from anterolateral arthroscopic portal of left ankle. She is known to have rheumatoid arthritis with no coagulation disorders. The patient had undergone arthroscopic synovectomy of left ankle done on 3 weeks previously, using 4.0-mm and 30 degree arthroscope under tourniquet control through anteromedial and anterolateral ports. There had been bleeding from the anterolateral port site post operatively but this had stopped quickly. The patient had been reviewed three times following the procedure by various doctors for similar problem who diagnosed as wound infection and discharged with assurance and oral antibiotics.

**Figure 1:**
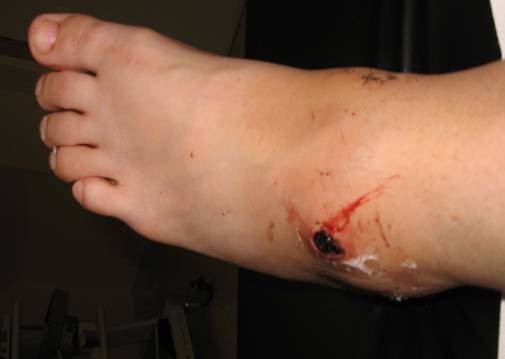
**Preoperative Photograph-Pulsatile pseudoaneurysm following ankle arthroscopy at antero lateral portal**.

Clinical examination revealed tender, indurated, an expansile pulsatile mass of over the anterolateral port site on the left ankle and pain with range of motion of ankle. There was a 1 cm wound with a central necrotic area over a swollen area measuring 3 × 6 cm (Figure 1). The wound had a persistent slow ooze of bright red blood and on palpation the swelling was pulsatile. Both the dorsalis pedis and the posterior tibial arteries were palpable. Sensation and perfusion of foot was normal. A duplex scan was performed which demonstrated triphasic flow in the dorsalis pedis and a pseudoaneurysm of the anterior tibial artery. Normal flow was reported in the anterior tibial artery from the upper calf to the site of the aneurysm, in which no flow seen. The patient had two units of blood transfusion, as she was breathless, pale and haemoglobin level was found to be 8.2 g/dl. Plain radiographs of the ankle did not demonstrate any abnormality, unlike osseous erosion due to pulsatile pseudoaneurysm at central to lateral side of distal tibia (Jang et al 2008).

**Figure 2:**
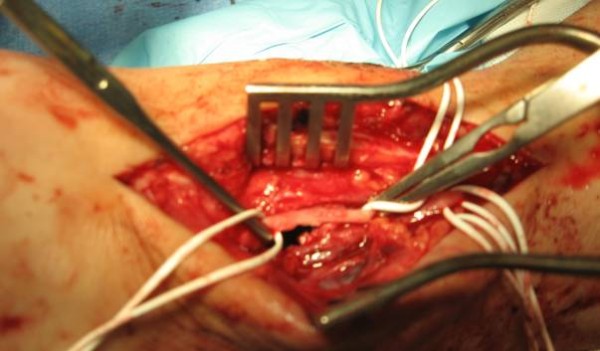
**Peroperative Photograph-serrated margin of anterior tibial artery**.

The patient was referred to the vascular team and underwent an urgent exploration. Intra-operatively, the findings were pulsatile mass with necrotic tissue, a moderately large cavity of pseudo aneurysm filled with fresh blood clots. The distal anterior tibial and dorsalis pedis arteries were isolated. The distal anterior tibial and proximal dorsalis pedis were found to be serrated and breached for 3 centimetres and in contact with sac. The damaged artery was identified proximally and distally and clamped (Figure 2). The clots from the sac were evacuated without compromising the blood supply to the foot. The patient made an uneventful recovery and was discharged to home after second look and closure of wound in 3 days. We reviewed her in outpatient clinic two weeks later, where she had made a good recovery with no residual symptoms.

## Discussion

Pseudo aneurysm is a rare complication of ankle arthroscopic surgery, with a reported incidence rate of 0.008% [6]. Due to the rarity of the complication, a high index of suspicion is required as it is easy to miss the diagnosis. The majority of cases of pseudoaneurysm involve the popliteal vessels following knee arthroscopy. This rarity of complication following ankle arthroscopy, resulted in treating our patient as infection. One should not forget anatomical variations of anterior tibial artery that has been described ranging from [7], 2.4% to [8], 12% and [9] and 1.5% to 3.3% by Darwish et al. Ferkel et al, has reported this complication using the anterocentral portal in a patient who underwent arthroscopic ankle arthrodesis. The same complication under the same circumstances was reported by Morgan et al. While reviewing the literature; we found that the postoperative time frame for development of pseudo aneurysm is quite variable. In the cases described by O'Farrell et al, and Mariani et al, the presentations were at 1 week, however Salgado et al, and [10] Jang et al. in 2 months. Although our case presented less than two weeks following the operation, there was a delay in diagnosis due to misdiagnosis until 3 weeks post procedure. The pseudoaneurysm formation occur earlier in patients with coagulopathies as in O Farrel et al (1997), Kotwal et al (2007), the case of Mariani et al and our case demonstrated that pseudo aneurysm may present earlier in patients with normal coagulation as well.

The mechanism of injury is not entirely clear; but looking at the ragged edge of artery could be due to iatrogenic injury during the removal of chronically inflamed synovium with a chondrotome, or during resection of the osteophytes or due to using the shaver. It is likely that bleeding and subsequent remodelling of the resultant haematoma eventually led to the formation of the pseudo aneurysm.

Various types of treatment methods are described to treat anterior tibial artery pseudo aneurysm ranging from non operative ultra sound guided compression therapy to operative treatment.

[10] Non operative ultra sound guided compression therapy described by Jang et al which compression is maintained three hours per day with three to four day interval between treatment and four treatments given in out patient department. We did not consider this option due to concern of infection which needs debridement, it is time consuming and our patient preferred operative treatment.

Operative treatment described were excision sac and end to end anastomosis by O Farrel 1997, excision of sac without arterial repair Salgado et al 1998, excision of sac and vein bypass graft Matian et al 2001, simple ligation of artery by Darwish et al 2004 and resection and reversed sephaneous vein interposition graft by Kotwal et al 2007. In our case debridement and simple ligation of anterior tibial artery done as patent Dorsalis, Posterior tibial artery with adequate foot perfusion.

The factors risk the injury of anterior tibial artery is anatomical course and variations, which makes it vulnerable. Yamada et al, reported that lateral deviation is present in 5.5% and medial deviation in 3.5% of the population. In this case lateral deviation of artery might be cause. We would like to mention some recommendations like careful dissection, Preoperative arterial mapping with a duplex scan or a handheld Doppler, as suggested by Darwish et al, is a useful for safer to approach arthroscopic surgery and with particular care in rheumatoid arthritis patients. In bony osteophytes, it is probably safer to perform open rather than arthroscopic surgery to minimize the risk.

## Conclusion

Pseudoaneurysm may present early or late in the postoperative period, and the clinician should be on the lookout for this rare potential complication to prevent missing this diagnosis. The surgeon should consider this rare complication as well as the anatomic variations of the artery when performing this procedure. Post operative ankle arthroscopy with not healing port wound and intermittent bleeding from port with or without osseous erosion of distal end of tibia, one should suspect pseudoaneurysm until proved otherwise.

## Consent

Written informed consent was obtained from the patient for publication of this case report and accompanying images. A copy of the written consent is available for review by the Editor-in-Chief of this journal.

## Competing interests

The authors declare that they have no competing interests.

## Authors' contributions

AR is the main author, who wrote the paper, JC performed editing and literature search. MG and DM are the patient consultants and senior authors.
